# Feeding Value Assessment of Substituting Cassava (*Manihot esculenta*) Residue for Concentrate of Dairy Cows Using an In Vitro Gas Test

**DOI:** 10.3390/ani11020307

**Published:** 2021-01-26

**Authors:** Yuhui Zheng, Yanyan Zhao, Shenglin Xue, Wei Wang, Yajing Wang, Zhijun Cao, Hongjian Yang, Shengli Li

**Affiliations:** State Key Laboratory of Animal Nutrition, College of Animal Science and Technology, China Agricultural University, Beijing 100193, China; zhengyuhui@cau.edu.cn (Y.Z.); LK20000403@163.com (Y.Z.); lin20010318@163.com (S.X.); wei.wang@cau.edu.cn (W.W.); yajingwang@cau.edu.cn (Y.W.); caozhijun@cau.edu.cn (Z.C.); yang_hongjian@sina.com (H.Y.)

**Keywords:** cassava residue, cumulative gas production, rumen fermentation

## Abstract

**Simple Summary:**

Cassava (*Manihot esculenta*) residue is a by-product of cassava processing. Although it contains residual nutrients, it is highly perishable. Decayed cassava residue pollutes the environment and leads to major losses in feed. If cassava residue could be utilized as a dairy cow feedstuff, these problems could be solved. Our study showed that cassava residue is a good alternative to concentrate in the feed of Holstein cows. Furthermore, our data demonstrate the efficacy of the application of cassava residue as a feed for dairy cows and could help solve the shortage of feed resources in China.

**Abstract:**

The feeding value of replacing concentrate with cassava (*Manihot esculenta*) residue in the feed of Holstein cows was confirmed using an in vitro gas test. The treatments consisted of 0% (control, CON), 5%, 10%, 15%, 20%, 25%, and 30% inclusion of cassava residue in fermentation culture medium composed of buffer solution (50 mL) and filtrated rumen fluid (25 mL). The parameters analyzed included the kinetics of gas production and fermentation indexes. Forty-eight hours later, there were no significant differences on in vitro dry matter disappearance (IVDMD), pH, and microbial crude protein (MCP) content among treatments (*p* > 0.05). However, the “cumulative gas production at 48 h” (GP_48_), the “asymptotic gas production” (A), and the “maximum gas production rate” (RmaxG) all increased linearly or quadratically (*p* < 0.01). The GP_48_ was significantly higher in the 25% treatment compared to the other treatments, except for the 30% (*p* < 0.01). The A was significantly larger in the 25% treatment compared to the other treatments, except for the 20% and 30% (*p* < 0.01). The RmaxG was distinctly larger in the 25% treatment compared to other treatments (*p* < 0.01); moreover, the “time at which RmaxG is reached” (TRmaxG) and the “time at which the maximum rate of substrate degradation is reached” (TRmaxS) were significantly higher in the 25% treatment than the CON, 20%, and 30% treatments (*p* < 0.01). Additionally, the content of ammonia-N (NH_3_-N) in all treatments showed linearly and quadratically decreases (*p* < 0.01), whereas total volatile fatty acid (VFA), iso-butyrate, butyrate, and iso-valerate contents changed quadratically (*p* = 0.02, *p* = 0.05, *p* = 0.01, and *p* = 0.02, respectively); all of these values peaked in the 25% treatment. In summary, the 25% treatment was associated with more in vitro gas and VFA production, indicating that this cassava residue inclusion level may be used to replace concentrate in the feed of Holstein cows. However, these results need to be verified in vivo.

## 1. Introduction

Cassava (*Manihot esculenta*) is a dominant crop grown in tropical and subtropical regions, and it is resistant to drought. Cassava production worldwide is estimated to be 276.7 million tons, and it provides a valuable food source for 105 countries [1,2,3]. Many factors limit the economic development of this crop, including its high cyanide content and short post-harvest shelf-life (its quality worsens within three days) [4,5]; nevertheless, cassava is still widely used in the production of starch, bioethanol, and other bioproducts such as feed, medicines, and biopolymers [3,6]. The contemporary expansion of cassava production has been mainly through starch extraction. In the harvest and processing season, plentiful residues are produced, most of which are lost as environmental pollution [7,8]. Cassava residue contains lots of calories and various contents of protein, ether extract, mineral substances, and vitamins, which make it highly nutritious compared with other tubers [9]. Thus, the application of cassava residues to the feed of Holstein cows could help reduce its environmental impact as well as the waste of nutrients.

One study demonstrated that dried residue after starch extraction could be used to feed dairy cows at around 100 days of lactation to replace more than 100% of corn [10]. One study showed that fermented cassava residue can be used as an energy source for beef cattle without impacting the digestibility as well as growth and meat performance [11]. Furthermore, Udchachon et al. [12] proved that the replacement of 46.8% of the concentrate with fermented cassava pulp feed for beef cattle improved the net profit of the animals by approximately USD 70 per head. Previous studies present in the bibliography mainly focused on the effects of cassava residue replacing concentrate on the performance of beef cattle. Nevertheless, the rapid development of China’s urbanization and increase in per capita income has led to a relative increase in milk consumption [13,14], which has increased demands for feed grains [15]; the supply of feed grains will be a major challenge to China’s food security [16]. Today, China is the sixth largest dairy producer in the world, with a total milk production of 33 million tons in 2019, accounting for about 3.9 percent of the world’s total milk production [17]. Therefore, if cassava residue can be developed as feed for dairy cows, it can not only turn waste into a valued product, but also greatly alleviate feed resource shortages in China. This study assessed the value of using cassava residue as a replacement of concentrate in the feed of Holstein cows using an in vitro gas test, and hopes to provide a basis for its utility as a feedstuff for dairy cows.

## 2. Materials and Methods

### 2.1. Material

Cassava residue and concentrate were offered by Jiuzheng Biotechnology Co. Ltd. (Beijing, China) and Zhongdi Animal Husbandry Technology Co. Ltd., respectively (Beijing, China). Cassava residue was obtained in Thailand after starch extraction and physical pressing. The analyzed nutrient composition of is shown in Table 1.

### 2.2. Rumen Fluid Collection

Approval for the experimental and animal care protocols was obtained from the Institutional Animal Care and Use Committee of China Agricultural University (Beijing, China) (No. AW09089102-1). Three Holstein cows (lactation 130 ± 20 days) were fed 2.5 kg of alfalfa hay, 1 kg of oat hay, 20 kg of whole corn silage, 2 kg of whole cottonseed, 4.2 kg of soybean meal, 4.5 kg of tablet corn, 1.5 kg of cornmeal, 1 kg of soybean hull, and 0.7 kg of a 1% premix daily. The rumen fluid was collected 1 h before feeding in the morning. The fluids were filtered via medical-grade cheesecloth (four layers) and stirred until evenly mixed. The blended rumen fluid was then moved into a vacuum bottle and taken to the laboratory within 1 h, warmed at 39 °C, and used as a culture medium.

### 2.3. Experimental Design

Seven treatments, each treatment with four replicates, were used. Specifically, cassava residue was substituted for concentrate at a ratio of 0% (control, CON), 5%, 10%, 15%, 20%, 25%, or 30% (air–dry basis). The composition of ingredients and nutrient contents in treatments is shown in Table 2. The concentrate used in this study contained 57.45% corn, 10.64% bran, 12.77% soybean meal, 15.96% cottonseed meal, 1.06% Ca(HCO_3_)_2_, 1.06% NaCl, and 1% premix (Contained vitamin E, 4000 IU; vitamin A, 6,000,000 IU; vitamin D, 100,000 IU; Cu, 2000 mg; Fe, 3000 mg; Mn, 2500 mg; Zn, 8000 mg; Se, 60 mg; I, 100 mg; and Co, 20 mg per kg).

### 2.4. In Vitro Batch Incubation

#### 2.4.1. In Vitro Batch Culture

The allocation of cultivation medium is shown in Table 3. CO_2_ gas was slowly and continuously injected into the medium for approximately 30 min until the color of the medium had transformed from red to pink and became colorless (pH ≈ 6.8). The prepared medium was sealed and warmed at 39 °C in thermal water. According to the design of the experiment, substrates (0.5 g) were placed in glass bottles (volume capacity: 120 mL). Freshly prepared and prewarmed medium (50 mL) as well as filtered and prewarmed rumen fluid (25 mL) were blended in each glass bottle and aerated with CO_2_ to exhaust the air. Additionally, the other four bottles containing no substrate were regarded as blanks. The bottles were sealed and immediately connected to an Automated Trace Gas Recording System (AGRS-III, China Agricultural University, Beijing, China) and cultured at 39 °C for 48 h to measure gas production [18]. The other bottles were treated in the same way: immediately placed in a thermostatic incubator and cultured for 3, 6, 12, and 24 h.

#### 2.4.2. Calculations

After cultivation, the bottles were removed from the system or taken out of the thermostatic incubator, and their contents were placed into pre-dried nylon bags with a pore size of 300 mesh to obtain supernatant samples. In vitro dry matter disappearance (IVDMD) was confirmed using the equation IVDMD = DM before incubation − DM after incubation (air–dry basis). Gas production was fitted by Equation (1) [19]:

GP_t_ = A/(1 + (C/t)^B^) (1)

where GP_t_ is the cumulative gas production (mL/g DM) at incubation time t (h), A is the asymptotic gas production (mL/g DM), B is a sharpness parameter determining the shape of the curve, Cis the time (h) at which half of “A” is reached, and t is the in vitro incubation time.

The time at which the maximum rate of substrate degradation is reached (TRmaxS, h), the maximum rate of substrate digestion (RmaxS, h), the time at which RmaxG is reached (TRmaxG, h), and the maximum gas production rate (RmaxG, mL/h) were calculated with A − C as Equations (2)–(5) [20]:

TRmaxS = C × (B − 1)^(1/B)^ (2)

RmaxS = (B × TRmaxS^(B−1)^)/(C^B^ + TRmaxS^B^) (3)

TRmaxG = C × ((B − 1)/(B + 1))^(1/B)^ (4)

RmaxG = (A × C^B^ × B × TRmaxG^−B−1^)/(1 + C^B^ × TRmaxG^(−B)^)^2^ (5)

#### 2.4.3. Analyses

After culture, the liquid supernatant filtered through nylon bags was stored at −20 °C to analyze ammonia-N (NH_3_-N), microbial crude protein (MCP) and volatile fatty acid (VFA) contents through bright blue colorimetry [21], Coomassie brilliant blue colorimetry [22], and gas chromatography [23], respectively. Moreover, pH was determined using a pH meter (Mettler Five Easy Plus series, Columbus, OH, USA). In detail, the NH_3_-N content was measured as follows: 50 μL supernatant samples of culture fluids were mixed with 2.5 mL phenol reagent contents of 50 mg sodium nitroprusside and 11 mL phenol solution per liter, 2.0 mL sodium hypochlorite reagent contents 5 g NaOH, 37.87 g Na_2_HPO_4_·7H_2_O and 50 mL 5.25% sodium hypochlorite per liter, warmed at 39 °C for 30 min, then tested under a wavelength of 595 nm; the MCP content was determined as follows: 1 mL supernatant samples of culture fluids centrifuged at 430× *g* for 5 min then 0.5 mL supernatant was centrifuged at 16,000× *g* for 15 min. The NaOH was added into the deposit and boiled for 10 min, 50 μL supernatant were blended with 150 μL Coomassie brilliant blue solution at 25 °C for 2 min, and a microplate reader was used to measure MCP content under a wavelength of 595 nm; the VFA content was measured as follows: 1 mL supernatant after centrifuged at 430× *g* for 10 min were blended with 0.2 mL 25% (w/v) meta-phosphoric acid solution contents 2-ethylbutyric acid for 30 min at 4 °C and then centrifuged at 16,000× *g* for 10 min and measured using gas chromatography (TP-2060, B.F.TianPu, Beijing, China).

### 2.5. Statistical Analysis

Data were fitted in the SAS 9.2 (SAS Institute, Carry, NC, USA) NLIN procedure for the kinetic parameters (“A”, “B”, “C”, “TRmaxG”, “RmaxG”, “TRmaxS”, and “RmaxS”). One-way ANOVA followed by Tukey’s multiple comparison test were used in this study. Linear and quadratic effects were confirmed by polynomial contrasts. Values of *p* ≤ 0.05 was defined as being significantly different.

## 3. Results

### 3.1. In Vitro Dry Matter Disappearance and Kinetic Parameters of Gas Production

Increasing cassava residue inclusion levels displayed no significant differences in IVDMD in all treatments at all culture time points analyzed (Table 4). However, after 24 h of culture, the IVDMD showed a linear increase (*p* = 0.02). Table 5 shows that GP_48_, A, and RmaxG increased linearly and quadratically (*p* < 0.01). Furthermore, the 25% treatment showed significantly higher GP_48_ compared with the other treatments, except for the 30% solution (*p* < 0.01). Kinetic parameter A was significantly larger in the 25% treatment than in other treatments, except for the 20% and 30% solutions (*p* < 0.01). B was significantly higher in the 25% treatment than the 5% and 10% solutions (*p* < 0.01). In the 15% treatment, C was significantly larger compared to the other treatments, except the 5% solution. TRmaxG was significantly higher in the 25% treatment than the CON, 20%, and 30% treatments (*p* < 0.01). RmaxG was significantly larger in the 25% treatment than the other treatments (*p* < 0.01). In the 10% treatment, TRmaxS was significantly longer than the CON, 20%, and 30% solutions (*p* < 0.01).

### 3.2. pH, Ammonia-N, and Microbial Crude Protein

After 12 h or 48 h of in vitro culture, the pH increased linearly with the ratio of cassava residue increasing (*p* = 0.04 and *p* = 0.04, respectively); after 24 h, the pH showed a quadratic change (*p* = 0.02). Additionally, the pH was significantly higher in the 10% treatment than CON and 5% after 12 h. No significant differences were observed among treatments after 3, 6, 24, and 48 h of in vitro culture (Table 6).

After 12 h of in vitro incubation, the NH_3_-N content was significantly lower in the 25% and 30% treatments than CON and 5% (*p* < 0.01) (Table 7); the NH_3_-N content in each treatment showed a linear and quadratic decrease (*p* < 0.01). No significant differences of the MCP content in all treatments were found at any incubation time point (Table 8).

### 3.3. Volatile Fatty Acid

The 15%, 20%, and 25% treatments had significantly more acetate than the 30% treatment (*p* = 0.04) (Table 9). The propionate and butyrate contents were significantly higher in the 25% treatment than CON, 5%, and 30% (*p* = 0.03). The iso-butyrate content was significantly higher in the 25% treatment than CON and 5% (*p* < 0.01). The iso-valerate content was lower in the 30% treatment in comparison with the other treatments, and this effect was significant except for the CON and 15% treatments (*p* = 0.05). The 25% treatment showed significantly higher total VFA than CON (*p* = 0.02). No significant differences were evident in acetate/propionate in different treatments. Additionally, total VFA, iso-butyrate, butyrate, and iso-valerate contents all showed quadratic changes (*p* = 0.02, *p* = 0.05, *p* = 0.01, and *p* = 0.02, respectively) and peaked in the 25% treatment.

### 3.4. Interaction between the Cassava Residue Inclusion Level and the In Vitro Incubation Time

The NH_3_-N content, MCP content, and pH all changed significantly when cultured for different periods (*p* < 0.01), whereas no significant influence of treatment × time was found. Furthermore, the NH_3_-N and MCP contents changed significantly as the levels of cassava residue inclusion increased (*p* < 0.01 and *p* = 0.04, respectively). The IVDMD did not significantly vary with treatment, time, or treatment × time (Table 10).

## 4. Discussion

### 4.1. Responses of Gas Production and Degradability to Cassava Residue Addition

Since the late 1970s, the in vitro gas test has been used more and more to determine feed digestive properties and fermentation kinetics [24]. In vitro gas production can provide an indicator of the digestibility of ruminant feeds in vivo [25]. Gas production was used to measure substrate degradation, especially carbohydrate fragments [26]. Previous studies have shown that gas production in the rumen of dairy cows is positively correlated with the starch content in the substrate [23]. The starch concentrates of cassava residue are approximately 49%. Therefore, as the proportion of cassava residue increases, the gas production also increases as a result of higher starch content in the fermentation medium. Gas production and the maximum gas production rate reflect microbial fermentation degree and substrate degradation rate. The 25% treatment showed significantly higher levels of these two indicators than CON, 20%, and 30% treatments, suggesting that the cassava residue enhanced the fermentation potential and accelerated the fermentation process [27]. Furthermore, the acid detergent fiber (ADF)content in the fermentation medium is a key factor affecting the in vitro degradability of the substrate. Higher ADF content corresponds to lower in vitro gas production [28]. Nevertheless, in our study, the ADF content in cassava residue was significantly higher than the concentrate, but as the proportion of cassava residue increased, the gas production also increased. This may have occurred because the addition of cassava residue altered the microbial composition in rumen fluid. Additionally, no significant differences were found between the IVDMD of each treatment group at each culture time. This result likely stemmed from the fact that feed degradation in the rumen is determined by nutrient decomposition by microorganisms, which can be affected by numerous factors, including rumen microbial composition and activity. Additional studies could be performed to verify cassava residue’s effect on microbial composition in dairy cows’ rumen. Smith et al. [29] indicated that the rumen degradation value of cassava residues was 88.5% after 12 h because of its high content of non-forage fibers as well as soluble carbohydrates. In this study, the IVDMD of each treatment group was greater than 80% when cultured for 24 h, indicating that the cassava residue had a higher degradation rate. Iqbal et al. [28] compared four typical subtropical forages and found that cassava residue was associated with the highest ruminal abundance of *Succinivibrio*, *Methanobrevibacter gottschalkii*, and *Entodinium*, which likely accounted for its higher degradation rate. The results obtained in the present study confirmed that using 25% cassava residue replacing concentrate of dairy cow did improve in vitro gas production, and beneficial response combined with the maximum gas production rate could be explained by our speculation that the addition of cassava residue could result in an improvement of rumen fermentation in Holstein cows.

### 4.2. In Vitro Rumen Fermentation Characteristics in Response to Cassava Residue Inclusion

Protein in feed is hydrolyzed into amino acids and peptides, and these small molecules are then decomposed by microorganisms to produce NH_3_-N. The NH_3_-N content is an indicator of protein degradation degree in rumen fermentation substrate [30]. Microbial protein is the main nitrogen source for dairy cows, and it can provide 60% to 80% of their protein requirements [31]. Previous studies have found that the NH_3_-N content in the rumen fluid was positively related to crude protein content in rumen fermentation substrate, but negatively correlated with the starch content. Higher starch content and lower crude protein content in rumen fermentation substrate leads to a lower rate of degradation by ruminal microbes [32]. Cassava residue contains a large amount of starch. Therefore, as the proportion of cassava residue increases, the starch content in the fermentation medium also increases, whereas the NH_3_-N content in the fermentation broth decreases linearly and quadratically. In addition, MCP synthesis is affected by various factors, such as microbial composition and activity. This could explain why there was no significant differences of MCP content in different treatments in this study. Therefore, in this study, NH_3_-N content decreased linearly and quadratically, whereas MCP content showed no significant differences. These results may be related to the composition and activity of ruminal microorganisms, which need further exploration on the effects of cassava residue inclusion levels.

An appropriate pH is required for the optimal growth of rumen microorganisms, and a low rumen pH (<6.4) for extended periods can negatively affect feed intake, lead to acidosis, and result in a decrease in milk fat in dairy cows [33]. In our study, the final pH values after in vitro culture for 48 h were distributed between 6.44–6.53, which were still within acceptable limits. Thus, cassava residue inclusion at all levels could provide a favorable environment for ruminal microorganisms. Previous studies have found that the protozoan population increases greatly when cultured with cassava residues with high levels of starches [34], and the function of ruminal protozoa is to swallow bacteria and starch particles in the rumen to maintain a stable pH [35]. These observations may explain why the pH was within a normal range in each treatment group. *Entodinium* was reported to be the dominant genus following in vitro culture with cassava residues using rumen fluids of dairy cows, and it had the highest starch intake rates than other protozoan species that were evaluated [36]. Additional studies could be carried out to assess the influence of cassava residues on the abundance of *Entodinium* in the rumen of Holstein cows. Primary end products of feed in rumen are volatile fatty acids, which could be easily assimilated and represented as the main form of energy for ruminants [37,38]. The content and ratio of VFAs reflect the metabolic condition of ruminal microbes, as well as whether the rumen microbial community primarily consists of fiber decomposers or amylase decomposers [39]. Acetate is produced from cellulose and hemicellulose [40], which are the main products of rumen microbial activity, and acetate is required for milk fat synthesis. Propionate is the main substrate for glucose synthesis during hepatic glycogenesis and is produced from lactose in milk [41]. The higher content of acetate and butyrate in 25% treatment and lower content in 30% treatment in this study indicated that although a higher content of cassava residue leads to higher crude fiber content in the fermentation substrate which could promote acetate and butyrate synthesis, excessive addition of cassava residue could inhibit the synthesis of acetate and butyrate in the rumen of dairy cows. Meanwhile, the similar result of propionate also indicated that although higher content of cassava residue could promote propionate synthesis due to higher content of starch in the rumen which usually produces a high proportion of propionate, excessive addition of cassava residue could inhibit the synthesis of propionate. Higher propionate, acetate, and butyrate content in the 25% treatment in this study indicated that cassava residue inclusion at this level may provide sufficient sources for gluconeogenesis and milk fat synthesis, respectively. Study [34] reported that *Succinivibrio* could effectively ferment carbohydrates into succinate and acetate, and it is positively associated with propionate and butyrate content; in this study, acetate, propionate, and butyrate contents were significantly higher in the 25% treatment compared to the control. Therefore, we speculate that the above results may be related to the fact that replacing dairy concentrate with 25% cassava residue can increase the relative abundance of *Succinivibrio* and thus promote the synthesis of related volatile fatty acids. Subsequent tests could be conducted to further investigate the microbial composition. In present study, the contents of propionate, butyrate, iso-butyrate and total VFA in the 25% treatment increased significantly compared to the control, and the contents of total VFA, iso-butyrate, butyrate and iso-valerate all changed quadratically and reached the peak value in the 25% treatment, which demonstrates that replacing concentrate with cassava residue could promote the synthesis of VFAs in rumen of Holstein cows.

## 5. Conclusions

In summary, this in vitro gas test indicated that the 25% treatment was associated with greater in vitro gas and VFA production, indicating that this cassava residue inclusion level may be used to replace concentrate in the feed of Holstein cows. However, these results need to be verified in vivo.

## Figures and Tables

**Table 1:** Analyzed nutrient content of the cassava residue and concentrate (air–dry basis, %).

Items	Concentrate	Cassava Residue
Dry Matter	96.94	96.51
Neutral Detergent Fiber	19.12	30.54
Acid Detergent Fiber	7.61	23.09
Crude Protein	20.60	8.46
Ether Extract	3.39	0.05
Calcium	0.52	0.91
Phosphorus	0.85	0.21
Ash	9.05	5.79

**Table 2:** Ingredient composition and nutrient contents in treatments (air–dry basis, %).

Items	Treatments						
	CON	5%	10%	15%	20%	25%	30%
Ingredients							
Concentrate	100.00	95.00	90.00	85.00	80.00	75.00	70.00
Cassava residue	0.00	5.00	10.00	15.00	20.00	25.00	30.00
Nutrient contents							
Dry Matter	96.94	96.92	96.90	96.88	96.85	96.83	96.81
Ether Extract	3.39	3.22	3.06	2.89	2.72	2.56	2.39
Crude Protein	20.60	19.99	19.39	18.78	18.17	17.57	16.96
Ash	9.05	8.89	8.72	8.56	8.40	8.24	8.07
Neutral Detergent Fiber	19.12	19.69	20.26	20.83	21.40	21.98	22.55
Acid Detergent Fiber	7.61	8.38	9.16	9.93	10.71	11.48	12.25
Phosphorus	0.45	0.44	0.43	0.41	0.40	0.39	0.38
Calcium	0.52	0.54	0.56	0.58	0.60	0.62	0.64

Treatments: Amount of cassava residue substituted for concentrate at ratio of 0% (CON), 5%, 10%, 15%, 20%, 25% and 30% (air–dry basis).

**Table 3:** Cultivation medium composition and addition order.

Addition Order	Component Solution	Volume (mL)
1	Distilled water	1200
2	Trace element solution A	0.3
3	Artificial saliva B	600
4	Constant element solution C	600
5	Resazurin solution D	3
6	Reducing agent solution	120
Total (mL)	2523.3	

Trace element solution A: 3.2 g of CaCl_2_·2H_2_O, 10 g of MnCl·4H_2_O, 1 g of CoCl_2_·6H_2_O, and 8 g of FeCl_3_·6H_2_O; volume of 100 mL with distilled water; Artificial saliva B: 35 g of NaHCO_3_ and 4 g of NH_4_HCO_3_; volume of 1000 mL with distilled water; Constant element solution C: 5.7 g of Na_2_HPO_4_, 6.2 g of KH_2_PO_4_, and 0.6 g of MgSO_4_·7H_2_O; volume of 1000 mL with distilled water; Resazurin solution D: 0.625 g of resazurin; volume of 100 mL with distilled water; Reducing agent solution: 0.625 g of Na_2_S·9H_2_O and 4 mL of 0.1 mol/L NaOH; volume of 100 mL with distilled water.

**Table 4:** Effects of cassava residue substituting for concentrate on in vitro dry matter digestibility (%).

In Vitro Incubation Time	Treatments							SEM	*p*-Value		
	CON	5%	10%	15%	20%	25%	30%		Treatment	Linear	Quadratic
3 h	36.33	38.14	39.19	39.15	38.92	41.42	39.01	0.01	0.63	0.19	0.31
6 h	50.62	48.40	47.98	49.96	47.68	50.30	51.86	0.01	0.97	0.67	0.61
12 h	68.17	66.84	67.08	67.64	69.46	70.41	69.18	0.01	0.19	0.02	0.06
24 h	81.88	83.32	81.80	84.00	83.74	82.13	84.08	0.01	0.98	0.49	0.79
48 h	83.41	85.99	83.85	81.08	82.96	84.28	84.29	0.01	0.46	0.96	0.68

Treatments: Amount of cassava residue substituted for concentrate at ratio of 0% (CON), 5%, 10%, 15%, 20%, 25% and 30% (air-dry basis); SEM: standard error.

**Table 5:** Effects of cassava residue substituting for concentrate on gas production and kinetic parameters.

Items	Treatments							SEM	*p*-Value		
	CON	5%	10%	15%	20%	25%	30%		Treatment	Linear	Quadratic
GP_48_ (mL/g)	108.32 ^d^	113.06 ^cd^	116.08 ^cd^	150.82 ^bcd^	149.64 ^bc^	188.58 ^a^	172.71 ^ab^	6.71	<0.01	<0.01	<0.01
A (mL)	114.11 ^c^	116.51 ^c^	115.11 ^c^	143.92 ^bc^	173.21 ^ab^	189.93 ^a^	170.94 ^ab^	6.75	<0.01	<0.01	<0.01
B (h)	1.19 ^abc^	1.07 ^c^	1.10 ^c^	1.11 ^bc^	1.28 ^abc^	1.45 ^ab^	1.15 ^abc^	0.04	0.03	0.33	0.44
C (h)	2.85 ^cd^	3.93 ^a^	2.85 ^cd^	4.34 ^a^	2.46 ^d^	2.92 ^bc^	3.30 ^b^	0.17	<0.01	0.99	0.61
TRmaxG (h)	0.47 ^cd^	0.85 ^abc^	1.21 ^a^	0.80 ^bcd^	0.43 ^d^	1.09 ^ab^	0.41 ^d^	0.07	<0.01	0.84	0.06
RmaxG (h)	25.03 ^cd^	20.77 ^d^	21.18 ^d^	21.95 ^d^	27.76 ^ab^	39.84 ^a^	33.00 ^b^	1.45	<0.01	<0.01	<0.01
TRmaxS (h)	1.14 ^b^	1.58 ^ab^	2.20 ^a^	1.51 ^ab^	0.83 ^b^	1.63 ^ab^	0.79 ^b^	0.11	<0.01	0.20	0.06
RmaxS (mL/h)	0.29	0.26	0.26	0.23	0.27	0.28	0.28	0.01	0.79	0.84	0.35

Treatments: Amount of cassava residue substituted for concentrate at ratio of 0% (CON), 5%, 10%, 15%, 20%, 25% and 30% (air–dry basis); SEM: standard error; GPt: the cumulative gas production (mL/g DM) at incubation time t (h); A: the asymptotic gas production (mL/g DM); B: a sharpness parameter determining the shape of the curve; C: the time (h) at which half of A is reached, and t is the in vitro incubation time; TRmaxS: The time at which maximum rate of substrate degradation is reached (h); RmaxS: the maximum rate of substrate digestion (/h); TRmaxG: the time at which RmaxG is reached (h); RmaxG: the maximum gas production rate (mL/h). ^a–d^: different superscripts indicate significant differences within a row (*p* ≤ 0.05).

**Table 6:** Effect of cassava residue substituting for concentrate on pH.

In Vitro Incubation Time	Treatments							SEM	*p*-Value		
	CON	5%	10%	15%	20%	25%	30%		Treatment	Linear	Quadratic
3 h	7.58	7.64	7.60	7.68	7.63	7.68	7.65	0.02	0.74	0.19	0.37
6 h	7.73	7.72	7.74	7.73	7.75	7.67	7.71	0.02	0.94	0.56	0.79
12 h	7.65 ^c^	7.67 ^bc^	7.73 ^a^	7.67 ^abc^	7.71 ^ab^	7.67 ^abc^	7.72 ^ab^	0.01	<0.01	0.04	0.09
24 h	7.48	7.54	7.55	7.55	7.59	7.57	7.52	0.01	0.13	0.28	0.02
48 h	6.44	6.45	6.49	6.48	6.50	6.52	6.53	0.02	0.65	0.04	0.13

Treatments: Amount of cassava residue substituted for concentrate at ratio of 0% (CON), 5%, 10%, 15%, 20%, 25% and 30% (air-dry basis); SEM: standard error; ^a–c^: means with different superscripts indicates significant difference within a row (*p* ≤ 0.05).

**Table 7:** Effects of cassava residue substituting for concentrate on ammonia-N content (mg/dL).

In Vitro Incubation Time	Treatments							SEM	*p*-Value		
	CON	5%	10%	15%	20%	25%	30%		Treatment	Linear	Quadratic
3 h	10.94	11.10	11.12	10.00	10.21	9.31	9.58	0.23	0.07	0.01	0.01
6 h	13.44	13.11	13.20	11.60	11.14	10.50	10.77	0.47	0.37	0.01	0.03
12 h	16.47 ^a^	16.57 ^a^	14.90 ^ab^	15.23 ^ab^	14.20 ^ab^	13.33 ^b^	13.01 ^b^	0.33	<0.01	<0.01	<0.01
24 h	24.44	24.72	22.56	22.63	21.40	20.47	20.18	0.84	0.68	0.01	0.04
48 h	31.65	32.25	30.13	31.10	29.25	28.59	25.71	0.80	0.38	0.02	0.04

Treatments: Amount of cassava residue substituted for concentrate at ratio of 0% (CON), 5%, 10%, 15%, 20%, 25% and 30% (air–dry basis); SEM: standard error; ^a,b^: different superscripts indicates significant differences within a row (*p* ≤ 0.05).

**Table 8:** Effect of cassava residue substituting for concentrate on microbial crude protein content (μg/mL).

In Vitro Incubation Time	Treatments							SEM	*p*-Value		
	CON	5%	10%	15%	20%	25%	30%		Treatment	Linear	Quadratic
3 h	170.33	170.67	178.61	167.51	180.06	174.41	175.97	2.32	0.23	0.20	0.44
6 h	195.85	197.27	199.08	188.07	204.81	191.96	198.41	5.00	0.99	0.97	0.99
12 h	218.15	220.31	219.51	228.78	232.38	224.54	228.56	2.89	0.55	0.08	0.18
24 h	205.13	207.65	209.89	204.69	215.16	214.81	211.16	2.58	0.83	0.25	0.51
48 h	174.70	174.33	179.03	172.59	182.26	169.42	175.03	5.86	0.74	0.83	0.85

Treatments: Amount of cassava residue substituted for concentrate at ratio of 0% (CON), 5%, 10%, 15%, 20%, 25% and 30% (air–dry basis).

**Table 9:** Effects of cassava residue substituting for concentrate on volatile fatty acid content and pattern after 48 h of cultivation (mmol/L).

In Vitro Incubation Time	Treatments							SEM	*p*-Value		
	CON	5%	10%	15%	20%	25%	30%		Treatment	Linear	Quadratic
Acetate	34.99 ^bc^	35.22 ^bc^	36.05 ^bc^	37.19 ^a^	37.23 ^a^	37.45 ^a^	33.74 ^b^	0.40	0.04	0.49	0.04
Propionate	15.73 ^b^	15.94 ^b^	16.28 ^ab^	16.17 ^ab^	16.55 ^ab^	17.95 ^a^	15.76 ^b^	0.22	0.03	0.29	0.34
Iso-butyrate	0.49 ^c^	0.55 ^bc^	0.70 ^abc^	0.71 ^abc^	0.80 ^abc^	0.87 ^a^	0.60 ^abc^	0.04	<0.01	0.09	0.02
Butyrate	7.13 ^b^	7.18 ^b^	7.28 ^ab^	7.53 ^ab^	7.54 ^ab^	7.78 ^a^	7.12 ^b^	0.07	0.03	0.10	0.05
Iso-valerate	1.84 ^ab^	1.98 ^a^	2.06 ^a^	1.92 ^ab^	2.10 ^a^	2.00 ^a^	1.70 ^b^	0.04	0.05	0.78	0.01
Valerate	2.68 ^ab^	2.44 ^ab^	2.24 ^ab^	2.37 ^ab^	2.45 ^ab^	2.92 ^a^	1.79 ^b^	0.12	<0.01	0.42	0.64
Acetate/Propionate	2.26	2.05	2.17	2.26	2.23	2.05	2.27	0.06	0.93	0.96	0.97
tVFA	51.94 ^b^	63.83 ^ab^	66.32 ^ab^	66.19 ^ab^	67.26 ^ab^	69.13 ^a^	60.70 ^ab^	1.92	0.25	0.17	0.02

Treatments: Amount of cassava residue substituted for concentrate at ratio of 0% (CON), 5%, 10%, 15%, 20%, 25% and 30% (air-dry basis); tVFA: total volatile fatty acids; SEM: standard error; ^a–c^: means with different superscripts indicate significant differences within a row (*p* ≤ 0.05).

**Table 10:** Effects of cassava residue substituting for concentrate on the in vitro feeding value.

Items	*p*-Value		
	Treatment	Time	Treatment × Time
IVDMD	0.45	0.43	0.47
NH_3_-N	<0.01	<0.01	0.99
MCP	0.04	<0.01	0.96
pH	0.97	<0.01	0.96

Treatments: Amount of cassava residue substituted for concentrate at ratio of 0% (CON), 5%, 10%, 15%, 20%, 25% and 30% (air–dry basis); Time: Cultured in vitro for 3, 6, 12, 24, or 48 h; In vitro dry matter disappearance (IVDMD, %) = DM before incubation − DM after incubation; NH_3_-N: Ammonia-N (mg/dL); MCP: Microbial protein (μg/mL).

## Data Availability

The data presented in this study are available on request from the corresponding author.
